# Circular RNA circUBE2J2 acts as the sponge of microRNA-370-5P to suppress hepatocellular carcinoma progression

**DOI:** 10.1038/s41419-021-04269-4

**Published:** 2021-10-22

**Authors:** Lu Zhang, Yachong Liu, Haisu Tao, He Zhu, Yonglong Pan, Pengcheng Li, Huifang Liang, Bixiang Zhang, Jia Song

**Affiliations:** 1 Hepatic Surgery Center, Tongji Hospital, Tongji Medical College, Huazhong University of Science and Technology, Wuhan, Hubei P.R. China; 2 Hubei Key Laboratory of Hepato-Pancreato-Biliary Diseases, Wuhan, Hubei P.R. China

**Keywords:** Tumour biomarkers, Small RNAs

## Abstract

Accumulating evidences indicate that circular RNAs (circRNAs), a class of non-coding RNAs, play important roles in tumorigenesis. However, the function of circRNAs in hepatocellular carcinoma is largely unknown. CircRNA microarray was performed to identify abnormally expressed circRNAs in HCC tissue samples. We conducted Kaplan–Meier survival analysis to explore the significance of circUBE2J2 in clinical prognosis. Then, we examined the functions of circUBE2J2 in HCC by cell proliferation, migration, and mouse xenograft assay. We identified miR-370-5P as a circUBE2J2-related microRNA by using biotin-labeled circUBE2J2 probe to perform RNA antisense purification (RAP) assay in HCC cells. The dual luciferase reporter assay and RNA pulldown assays were employed to verify the relationships among circUBE2J2, miRNA-370-5P, and KLF7. Microarray analysis and qRT-PCR verified a circRNA termed circUBE2J2 that was downregulated in HCC. Kaplan–Meier survival analysis showed that downregulated circUBE2J2 was correlated with poorer survival. CircUBE2J2 expression in HCC cells was selectively regulated via luciferase reporter assays; circUBE2J2 and KLF7 were observed to directly bind to miR-370-5P. Furthermore, knockdown of circUBE2J2 in HCC could downregulate KLF7, the target of miR-370-5P, thus promoting the proliferation and migration of HCC cells. Then the related experiment suggested that circUBE2J2 could regulate the expression of KLF7 by sponging miR-370-5p. In summary, we infer that circUBE2J2 may act as a competing endogenous RNA (ceRNA) to regulate KLF7 expression through sponging miR-370-5P and play a regulatory functions in HCC. CircUBE2J2 may be a diagnostic biomarker and potential target for HCC therapy.

## Introduction

Primary liver cancer ranks sixth among the most commonly diagnosed cancers in the world and is also the third leading cause of cancer death [1]. Hepatocellular carcinoma (HCC) accounts for 75–85% of primary liver cancers. Chronic hepatitis B virus (HBV) or hepatitis C virus (HCV) infection, aflatoxin-contaminated food, and heavy alcohol consumption are the major risk factors for HCC [2]. Patients with HCC have a high frequency of metastasis and recurrence and a poor prognosis [3, 4]. Over the past few decades, great efforts have been made to treat HCC clinically [5, 6]. However, since its pathogenesis at the molecular level is not clear, there is a lack of specific targets. Therefore, it is necessary to search for new biomarkers for the treatment of HCC. Circular RNAs (circRNAs) are a covalently enclosed endogenous biomolecules, which has tissue specificity and cell specificity in eukaryotes, and are produced after being regulated by specific *cis*-acting elements and *trans*-acting factors. Most circRNAs are abundant and evolutionarily conserved. Many circRNAs regulate protein function or self-translation by acting as sponges for microRNAs or proteins [7]. CircRNAs are widely expressed in mammals and belong to a class of non-coding RNAs [8]. CircRNAs have recently been shown to be involved in neuronal development, regulation of cellular function, immune regulation, pathogenesis of heart failure, and the development of diabetes [9–12]. Certain types of circRNAs have been found to be significantly dysregulated in gastric cancer, bladder cancer, and triple-negative breast cancer, and these deregulated circRNAs are thought to be involved in the development of cancer [13–15]. These findings indicate that circRNAs may become a new type of potential biomarker and therapeutic target for cancer; however, clarifying the dysregulated circRNAs and identifying their functions are still an ongoing process in cancer research. MicroRNAs (miRNAs) are endogenous, non-protein coding, single-stranded 19–25-nucleotide RNAs that play a vital role in the process of cancer [16]. It is reported that RNAs can act as competitive endogenous RNAs (ceRNAs) to co-regulate each other by competing for shared microRNAs [17, 18].

In our research, we analyzed the expression profile of circRNAs in HCC tissues and found that circRNA circUBE2J2 (hsa_circ_0009246) is significantly downregulated in HCC tissues compared with paracancer HCC tissues, which was closely related to the prognosis of HCC patients. We identified that circUBE2J2 may function as the sponge of oncogenic miR-370-5P to upregulate KLF7 expression and consequently suppress HCC progression. Therefore, the reduction of circUBE2J2 may be used as a prognostic biomarker and as a potential therapeutic target for HCC patients.

## Materials and methods

### Tissues and cell lines

A total of 75 pairs of HCC tissues and adjacent normal tissues were collected from HCC patients who were treated at Tongji Hospital in Wuhan, China, between January 2015 and December 2018. All patients have signed an informed consent to donate tissue samples for biomedical research approved by the ethics committee of Tongji Hospital. HCC diagnosis was confirmed by pathologists and their clinical stages were determined, according to the BCLC classification.

HCC patients with the following conditions were excluded: (1) patients ≤18 or ≥70 years of age; (2) patients with a history of preoperative anticancer radiotherapy or chemotherapy, biological, immune, and traditional Chinese medicine; (3) patients with incomplete postoperative follow-up data; (4) patients with a history of another organ malignancy or systemic immune disease.

The human liver cancer cell lines MHCC97H were obtained from the Liver Cancer Institute of Fudan University; HepG2 and HLF were purchased from China Center for Type Culture Collection (CCTCC, Wuhan, China). HEK293T cells were deposited in the Hepatic Surgery Center, Tongji Hospital. All cell lines were cultured in DMEM with 10% FBS (Gibco) and incubated in 5% CO_2_ at 37 °C.

### Microarray analysis

Five pairs of HCC samples (tumor tissues and matched non-tumor tissues) were analyzed by Arraystar Human circRNA Array. The circRNAs chip (Arraystar Human circRNAs chip, ArrayStar) containing probes specific for human circular RNAs splicing sites was used. Exogenous RNAs developed by ERCC (External RNA Controls Consortium) were used as controls.

### Quantitative PCR

TRIzol reagent (Invitrogen, USA) was used to extract the total RNAs of tissues and cell lines. Cytoplasmic and nuclear RNA isolation were performed with PARIS™ Kit (Invitrogen, USA) following the manufacturer’s instruction. RNA concentration was measured by Nonodrop and each paired sample was adjusted to the same concentration. The RNA samples (2–3 µg each) were reversely transcribed into cDNA by using the HiScript^®^II Q RT SuperMix for qPCR (Vazyme Biotech Co, Ltd). For miRNA, reverse transcriptions were performed using the Mir-X miRNA First-Strand Synthesis Kit (Takara, Japan). Real-time qPCR was performed as described previously [19]. U6 and GAPDH were used as endogenous controls for the detection of circRNA, miRNA, and mRNA expression, respectively. Relative quantification analysis was performed using the comparative CT (2−ΔΔCT) method. Each experiment was repeated independently three times. The primer sequences are available in Supplementary Table1.

### Actinomycin D assay

HCC HepG2 and HLF cells were exposed to 2 μg/mL actinomycin D (Sigma-Aldrich, St. Louis, MO, USA) or DMSO (Mock) (Sigma-Aldrich) as the negative control at indicated time point. Then the cells were harvested, and total RNA was extracted. The stability of circUBE2J2 and UBE2J2 mRNA was analyzed using quantitative reverse transcriptase PCR (qRT-PCR).

### Transfection experiment

Small hairpin RNAs (shRNAs) of circUBE2J2 plasmids pLKD-CMV-circUBE2J2 targeting to the junction region of circUBE2J2 sequence and its negative control were synthesized by ObiO Biotech (Shanghai, China). The plasmid pLO5-circUBE2J2 and its negative control were obtained from Geneseed Biotech (Guangzhou, China). MiRNA-370-5p mimics, miRNA-370-5p inhibitor, and the corresponding negative control were synthesized by Sangon Biotech (Shanghai, China). The relevant miRNA-derived lentiviruses, plasmids for expression of KLF7, were purchased from Genechem (Shanghai, China). SiKLF7 (si1-KLF7: GCACGGTGACGTTGAAACT;

si2-KLF7: CTCTCAGCTCCGTAAAGGT) and the corresponding negative control were purchased from RiboBio (Guangzhou, China). Lipofectamine™ 3000 (Invitrogen,USA) was used as the transfection reagent following the standard process.

### Cell proliferation assay

Cell proliferation ability was assessed by Cell Counting Kit-8 (Beyotime Institute of Biotechnology). Indicated (1000–2000) cells were seeded in 96-well plates. CCK-8 was added to the plates for 1–2 h to determine the OD_450_ using a microplate reader (Bio-Tek Instruments, USA). The result was then calculated using GraphPad Prism, version 6.0.

For colony formation assays, 1000–2000 cells were plated in six-well plates. Medium was replaced with fresh culture medium every 2 days. After 10–14 days, the plates were stained with 1% crystal violet (Sigma-Aldrich) and photographed. Colonies were counted and analyzed using the Alpha Innotech Imaging system (Alphatron Asia, Singapore).

We studied DNA synthesis and cell proliferation using the EDU assay kit (RiboBio, Guangzhou, China). We seeded 8000–10,000 HCC cells into 96-well plates. The next day, EDU solution (100 μm) was added to the culture dish and incubated for 2 h. The cells were then fixed with 4% formalin at room temperature for 30 min. Then, the cells were permeabilized with 0.5% Triton X-100 for 10 min, and Apollo reaction solution (200 μL) was added, nucleus were stained with 1× Hoechst33342 solution. Representative images were captured with a Nikon microscope (Tokyo, Japan), and positive cells were counted by ImageJ pro plus6.0 software.

### Transwell and wound-healing assays

We suspended HCC cells in 250 μL of serum-free medium and inserted them into a upper chamber of Transwell plates with 8-μm pores (Corning Costar, Corning, NY, USA). HCC cells (2 × 10^4^ HLF cells, 3 × 10^4^ 97H cells, and 2 × 10^4^ HepG2 cells) were cultured in serum-free medium in the top chamber. We also placed 600 μL medium containing 10% FBS in lower chamber as chemoattractant. After incubation for 1 day, the cells on the upper surface of the top chamber were removed with a cotton swab, and the invaded cells on the bottom surface of the top chamber were stained with 0.1% crystal violet solution and photographed under a light microscope, and then counted them in three random fields of view (×100).

For wound-healing assays, transfected cells were inoculated into six-well plates. A linear scratch wound was created with a 10-μL pipette tip after cells reaching confluence. The cells were then washed with PBS and incubated in medium without FBS. We observed and photographed wound closure under a microscope during 0–48 h after wounding.

### RNA antisense purification (RAP) assay

RAP assay was performed using a RAP Kit (BersinBio, Guangzhou, China) according to the manufacturer’s protocol. Biotin-labeled circUBE2J2 probes (biotin-5′- CCTCTTACTGCTGGTGCTGCTCATCTCCACGACTTCAGGAAATAATTC-3′-biotin) and negative control probes were synthesized by Sangon Biotech (Shanghai, China). Biotin-labeled miR-370-5P and negative control probes were synthesized by RiboBio (Guangzhou, China).

### Anti-Ago2 RNA-binding protein immuno-precipitation (RIP) assay

Rabbit anti-Ago2 IgG was purchased from Abcam (ab32381). Magna RIP™ RNA-Binding Protein Immunoprecipitation Kit (Millipore, USA) was used to enrich Ago2 binding RNA. The enriched RNA was subjected to qRT-PCR. 2−ΔCT was calculated and normalized to the 2−ΔCT of 10% input.

### Dual luciferase assay

Luciferase reporter vector with the full length of the 3′-UTR of circUBE2J2 or KLF7 and the mutant version were constructed. Luciferase reporter vector with miR-370-5P mimics or mimics nc were transfected into HEK293T or HCC cells. Two days later, the cells were lysed and their luciferase activities were measured by using the dual luciferase reporter assay system (Promega).

### Western blotting

Western blot analysis was performed using standard procedures. Briefly, total proteins were extracted and separated by 10% sodium dodecyl sulfate polyacrylamide gel electrophoresis (SDS-PAGE) and transferred onto a polyvinylidene difluoride (PVDF) membrane (Millipore, USA). After blocking with 5% bovine serum albumin (BSA), the membranes were probed with primary antibodies for one night at 4 °C and detected with horseradish peroxidase (HRP)-conjugated secondary antibodies (1:5000, AP308P, Sigma-Aldrich), followed by visualization by using enhanced chemiluminescent reagents. The primary antibodies included KLF7 Polyclonal Antibody (1:500, 24693-1-AP, Proteintech, China), anti-occludin (1:1000, BM4832, Boster, BioEngineering Company, Wuhan, China), anti-N-cadherin (1:1000, 22018-1-AP, Proteintech, China), anti-Vimentin (1:1000, D21H3, CST), and anti-GAPDH (1:5000, 60004-1-Ig, Proteintech, China). The experiment was repeated three times.

### Flow cytometric analysis of cell cycle progression

For the cell cycle assay, indicated cells were harvested for the following steps. Cells were fixed in 70% ethanol overnight at 4 °C. Then, the fixed cells were resuspended in staining solution (Beyotime, Shanghai) and were incubated for 30 min at 4 °C. A total of 1 × 10^6^ cells per sample were analyzed for cell cycle distribution on a FACS Aria Cell Cytometer (BD Biosciences, San Jose, CA, USA).

### Fluorescence in situ hybridization (FISH)

FISH assay was performed to detect the location of circUBEJ2 and miR-370-5P in HCC cells. Cy3-labeled circUBE2J2 probes were synthesized by Sangon Biotech (Shanghai, China) and FAM-labeled miR-370-5P were ordered from RiboBio (Guangzhou, China). Hybridization steps were performed using Fluorescent In Situ Hybridization Kit (RiboBio, China) according to the manufacturer’s instructions. We counterstained nuclei with 4,6-diamidino-2-phenylindole (DAPI). Confocal images were acquired on a laser scanning confocal microscope (LSM710, Carl Zeiss, Germany). The sequence of circUBE2J2 probe for FISH was 5′-CCTCTTACTGCTGGTGCTGCTCATCTCCACGACTTCAGGAAATAATTC-3′-Cy3. The sequence of miR-370-5P probe for FISH was FAM-5′-CAGGTCACGTCTCTGCAGTTAC-3′.

### In situ hybridization (ISH)

The expression level of circUBE2J2 in tissues was evaluated by ISH using specific digoxin-labeled circUBE2J2 probe on HCC tissue. The probes were synthesized by Sangon Biotech (Shanghai, China). The sequence of circUBE2J2 probe for ISH was Digoxin-5′- CCTCTTACTGCTGGTGCTGCTCATCTCCACGACTTCAGGAAATAATTC-3′-Digoxin.

### Immunohistochemistry (IHC) staining

We fixed tumor tissue samples in 10% formalin and embedded them in paraffin. We stained tissues sections with Ki67 (1:200, Abcam, USA) to explore proliferation. We examined sections and photographed images under a light microscope.

### Xenograft tumor model

All animal care and experiments were carried out in accordance with the National Institutes of Health Guidelines for the Care and Use of Laboratory Animals and approved by the Ethics Committee of Tongji Hospital, HUST. Four-week-old male nude mice were purchased from Vital River BioScience (Beijing, China) and maintained in specific-pathogen-free conditions. (The nude mice were randomly grouped, five in each group). To generate the subcutaneous tumor model in nude mice, 1 × 10^6^ cells in 100 μL serum-free DMEM were injected into the axillary region of mice. For the orthotopic model, 1 × 10^6^ cells in 30 μL serum-free DMEM were injected into the left hepatic lobe of nude mice. After 5 weeks, the mice were sacrificed and the tumor tissues were detected for tumor weight, volume, and IHC staining. Tumor volumes were calculated according to the following equation: *V* (volume, mm^3^) = *π*/6 × *L* (length, mm) × *W*^2^ (width, mm^2^).

### Statistical analysis

Statistical analysis was performed with SPSS software19.0 or GraphPad Prism 6.0. In brief, the values are expressed as the mean ± standard deviation (SD). Data were compared using two-tailed Student’s *t*-test or one-way ANOVA test. Pearson correlation coefficient was used to analyze the linear correlations. The categorical data were analyzed by chi-square or Fisher’s exact tests. The cumulative recurrence and survival rates were analyzed using Kaplan–Meier’s method and the log-rank test. Univariate Cox proportional hazard regression analysis and multiple Cox regression analysis were carried out. The *survminer* R package was used to calculate the optimal cut-off value, and then the patients were divided into high- and low-risk cohorts. By using *survival* R package, the Kaplan–Meier survival curve (KM curve) could be used to compare prognostic significance. *P* < 0.05 was considered statistically significant: *p < 0.05, **p < 0.01; NS no significance.

## Results

### CircRNA expression profile in TNBC and decreased circUBE2J2 expression associated with more aggressive clinic features of HCC patients

**Fig. 1:**
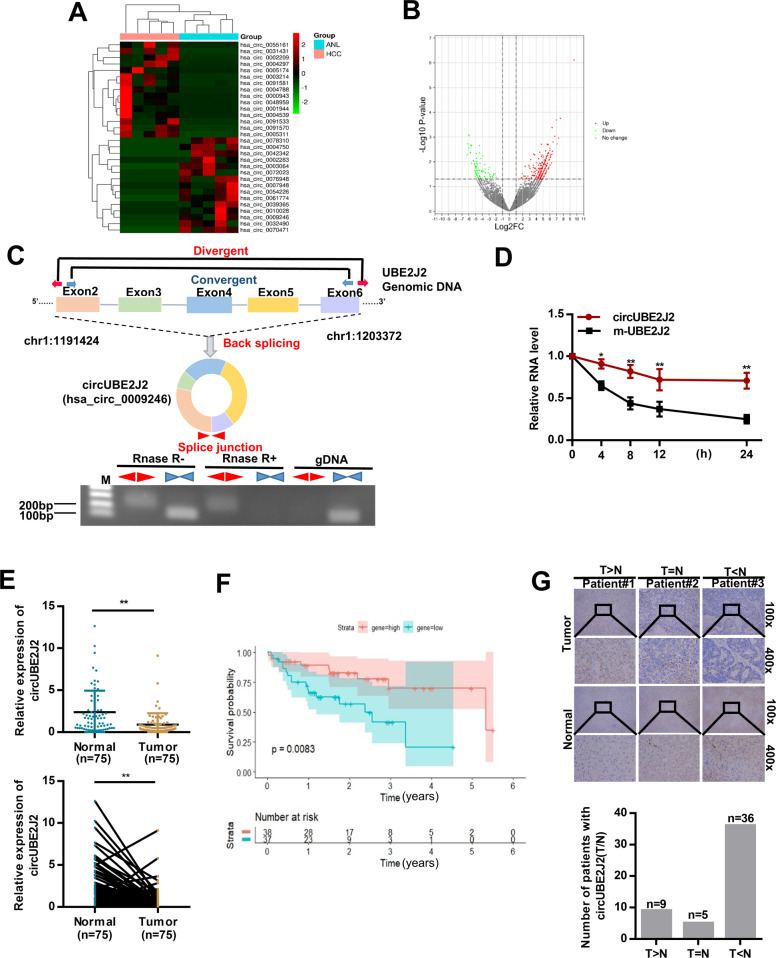
Down-regulated circUBE2J2 in HCC tissues and prognostic significance. **A** The cluster heat map showed the differentially expressed circRNAs in HCC tissues compared with those in adjacent nontumor tissues. Red color indicates high expression level, and green color indicates low expression level. **B** Volcano plots illustrate that among significantly different expressed circRNAs in HCC tissues relative to normal tissues. The green, red, and black points represent downregulated, upregulated, and no statistically significant difference circular RNAs (circRNAs), respectively. *x*-axis: log2 ratio of circRNA expression levels between normal and tumor tissues. *y*-axis: the *P* value (−log10 transformed) of circRNAs. **C** Upper panel: illustration of the annotated genomic region of *UBE2J2*, the putative different RNA splicing forms, and scheme illustrating the production of circUBE2J2. Convergent (blue) and divergent (red) primers were designed to amplify the linear or back-splicing products. Lower panel: total RNA from HepG2 cells with or without RNase-R treatment were subjected to polymerase chain reaction (PCR). **D** qRT-PCR analysis of circUBE2J2 and mUBE2J2 RNA after treatment with Actinomycin D at the indicated time points in HCC cells. **E** qRT-PCR detection show the differential expression of circUBE2J2 in 75 paired HCC tissues. **F** CircUBE2J2 expression among 75 patients was analyzed for survival probability*.* **G** The expression of circUBE2J2 was analyzed by in situ hybridization on HCC tissue. The representative photomicrographs of circUBE2J2 level with high, equal, and low in HCC tumors, as compared with normal tissues, were shown. The altered level of circUBE2J2 between HCC and non-tumor tissues in 50 HCC patients were summarized (*n* = number of cases). Data are presented as mean ± SD. *p < 0.05, **p < 0.01; NS no significance.

Five pairs of HCC tissue samples (five pairs of HCC tissue and five pairs of matched non-tumor liver tissue) were analyzed by using CircRNA microarray to observe the expression profile of CircRNA in HCC tissues. According to the classification of expression intensity, compared with non-tumor tissues, several differentially expressed circRNAs in HCC tumor tissues are shown in Fig. 1A and the variation of circRNAs expression was revealed in the volcano plots (Fig. 1B). The result showed that 233 circRNAs were upregulated and 86 circRNAs were downregulated with fold change greater than 2, *p* < 0.05 and FDR < 0.05. The top 20 circRNAs up-regulated or down-regulated in HCC tumor tissues are shown in Table 1.

**Table 1:** top 20 up-regulated or down-regulated circRNAs in five pairs of HCC tissues.

Acc ID	log2FC	*P* value	HCC1	HCC2	HCC3	HCC4	HCC5	ANL1	ANL2	ANL3	ANL4	ANL5
hsa_circ_0072023	–6.23944191	0.005254189	0	0	0	0	0	10	27	0	0	21
hsa_circ_0007948	–6.14551502	0.001897676	0	0	0	0	0	0	0	14	32	4
hsa_circ_0042342	−6.02411099	0.002788803	0	0	0	0	0	19	16	11	9	0
hsa_circ_0054226	−6.01709313	0.000819392	0	0	0	0	0	0	2	10	24	10
hsa_circ_0032490	−5.93916462	0.000856059	0	0	0	0	0	8	5	18	0	13
hsa_circ_0002283	−5.76348133	0.004341122	0	0	0	0	0	8	21	0	3	0
hsa_circ_0009246	−5.72997053	0.004239146	0	0	0	0	0	5	0	34	13	11
hsa_circ_0003064	−5.69595329	0.002106758	0	0	0	0	0	9	16	7	3	9
hsa_circ_0070471	−5.63427962	0.002170006	0	0	0	0	0	2	10	14	9	15
hsa_circ_0076948	−5.58282287	0.011523781	0	0	0	0	0	0	0	20	22	14
hsa_circ_0091533	6.063545663	0.006747943	54	0	11	64	0	0	0	0	0	0
hsa_circ_0005174	6.078787551	0.006364455	13	69	0	6	0	0	0	0	0	0
hsa_circ_0091581	6.10882153	0.00538621	212	0	0	29	134	0	0	5	0	0
hsa_circ_0055161	6.269965358	0.011029514	13	0	0	50	95	0	0	0	0	0
hsa_circ_0091570	6.298485869	0.004974971	52	0	37	22	0	0	0	0	0	0
hsa_circ_0005311	6.345520095	0.000413961	27	0	22	16	11	0	0	0	1	0
hsa_circ_0031431	6.397499608	0.003766047	23	0	0	33	25	0	0	0	0	0
hsa_circ_0002209	6.469490879	0.001604919	4	0	60	69	16	0	0	0	0	0
hsa_circ_0003214	6.737478747	0.00093628	77	0	6	20	39	0	0	0	0	1
hsa_circ_0048959	6.990062309	0.00020524	103	0	19	2	8	0	0	1	0	0

Next, we performed qRT-PCR to verify the expression of the top 10 downregulated circRNAs in HCC tissues and matched non-tumor tissue samples from eight patients, and the expression of the three circRNAs with the largest changes were presented in Additional file 1 Supplementary Fig. 1a. Among them, we found that the expression of circUBE2J2(hsa_circ_0009246) was significantly decreased. Thus, we chose circUBE2J2 for subsequent research. According to human reference genome (GRCh37/hg19), we further assumed that hsa_circ_0009246, located at chr1: 1191424–1203372, is derived from exons 2, 3, 4, 5, and 6 of the gene UBE2J2 (ubiquitin-conjugating enzyme E2 J2) which is located on chromosome 1p36.33 (Additional File 1 Supplementary Fig. S1b). Therefore, we termed hsa_circ_0009246 as “circUBE2J2”. In order to verify that circUBE2J2 is an endogenous circular RNA formed by exons 2, 3, 4, 5, and 6 of the UBE2J2 gene, we designed divergent and convergent primers (Supplementary Table) that specifically amplified the back-spliced or canonical forms of UBE2J2, respectively (Fig. 1C, upper panel). These primers cannot amplify any other products in genomic DNA. The convergent primer (red) detected circUBE2J2 by polymerase chain reaction (PCR) after reverse transcription, which can resist RNase-R digestion [20]. In contrast, PCR products amplified by the convergent primers (blue) using linear UBE2J2 mRNA as a template disappeared after RNAse-R digestion (Fig. 1C, lower panel). Furthermore, treatment HCC cells with actinomycin D [21, 22], an inhibitor of transcription, revealed that the half-life of circUBE2J2 transcript was more stable in comparison to UBE2J2 mRNA (Fig. 1D). Together, these data confirmed the characteristics of circUBE2J2 as a circRNA.

Next, we explored the potential clinicopathological implications of circUBE2J2 in HCC. Seventy-five pairs of surgical HCC and adjacent nontumor liver tissues were obtained, and the demographic and clinical characteristics of those patients are shown in Table 2. The results demonstrate that HCC patients with circUBE2J2^low^ had larger tumor sizes (*p* = 0.028), encapsulation invasion (*p* = 0.043), and multiple tumors (*p* = 0.020) compared with those in HCC patients with circUBE2J2 high. Multivariate analysis identified CircUBE2J2 expression as an independent predictor for overall survival (OS) (Table 3). CircUBE2J2 expression levels were examined in tissues of 75 HCC patients using qRT-PCR.

**Table 2:** Correlation between circUBE2J2 and clinicopathological characteristics in 75 HCCs.

Variables	Circular (low)	Circular (high)	*P* value
Age, years, ≥55/<55	16/21	16/22	0.921
Sex, male/female	18/19	23/15	0.302
AFP, ng/Ml, <55/≥55	15/22	14/24	0.742
ALT, U/L, <40/≥40	19/18	21/17	0.734
AST, U/L, <40/≥40	14/23	20/18	0.198
Liver cirrhosis, yes/no	20/17	21/17	0.916
HBsAg, negative/positive	10/27	11/27	0.853
HCVAb, negative/positive	33/4	32/6	0.736
Tumor size, cm, <5/≥5	13/24	23/15	0.028*
Encapsulation invasion, absent/present	20/17	29/9	0.043*
Vascular invasion, no/yes	18/19	19/19	0.907
Tumor number, single/multiple	28/9	36/2	0.020*
TNM staging, I–II/III–IV	21/16	19/19	0.558

*P < 0.05, statistically significant.

**Table 3:** Univariate and multivariate analysis for overall survival.

	Univariate analysis		Multivariate analysis	
Variables	Hazard ratio	*P*	Hazard ratio	*P*
Age, years, ≥55/<55	0.9968	0.9937		NA
Sex, male/female	1.0369	0.9278		NA
AFP, ng/Ml, <55/≥55	1.6103	0.2633		NA
ALT, U/L, <40/≥40	0.7349	0.4403		NA
AST, U/L, <40/≥40	1.4602	0.3502		NA
Liver cirrhosis, yes/no	0.9192	0.8306		NA
HBsAg, negative/positive	0.9346	0.8748		NA
HCVAb, negative/positive	0.9873	0.9834		NA
Tumor size, cm, <5/≥5	3.4156	0.0049	2.8834	0.0176
Encapsulation invasion, absent/present	1.7245	0.1915		NA
Vascular invasion, No/Yes	0.8355	0.6518		NA
Tumor number, single/multiple	2.7555	0.0491		NA
TNM staging, I–II/III–IV	0.8016	0.5784		NA
Circular, low /high	0.3337	0.0117	0.4106	0.0451

*NA* not adopted, *AFP* alpha-fetoprotein, *HBsAg* hepatitis B surface antigen l, Cox proportional hazards regression model.

The results showed that circUBE2J2 expression was significantly decreased in 76% (57 of 75) HCC tissues (Fig.1E). Based on the median of circUBE2J2 expression in tumor tissues, patients were divided into circUBE2J2 low expression and circUBE2J2 high expression. Patients with circUBE2J2 expression levels equal to or greater than the average circUBE2J2 expression level were defined as “gene high” group and the rest as “low gene”. The hypothesis test results of the two survival curves with high expression and low expression circUBE2J2 showed that the difference between the two curves was statistically significant (Fig. 1F). CircUBE2J2 expression was determined using ISH analysis on HCC tissue. As shown in Fig. 1G, circUBE2J2 expression was also significantly decreased in HCC tissues as compared to that in matched non-tumor tissues. These results revealed that circUBE2J2 might play a vital role in tumor progression of HCC. The abnormal expression of circUBE2J2 in HCC tissues inspired us to pay attention to the biological significance of circUBE2J2 in the progression of HCC.

### Decreased circUBE2J2 promotes the malignant behaviors of HCC cells

**Fig. 2:**
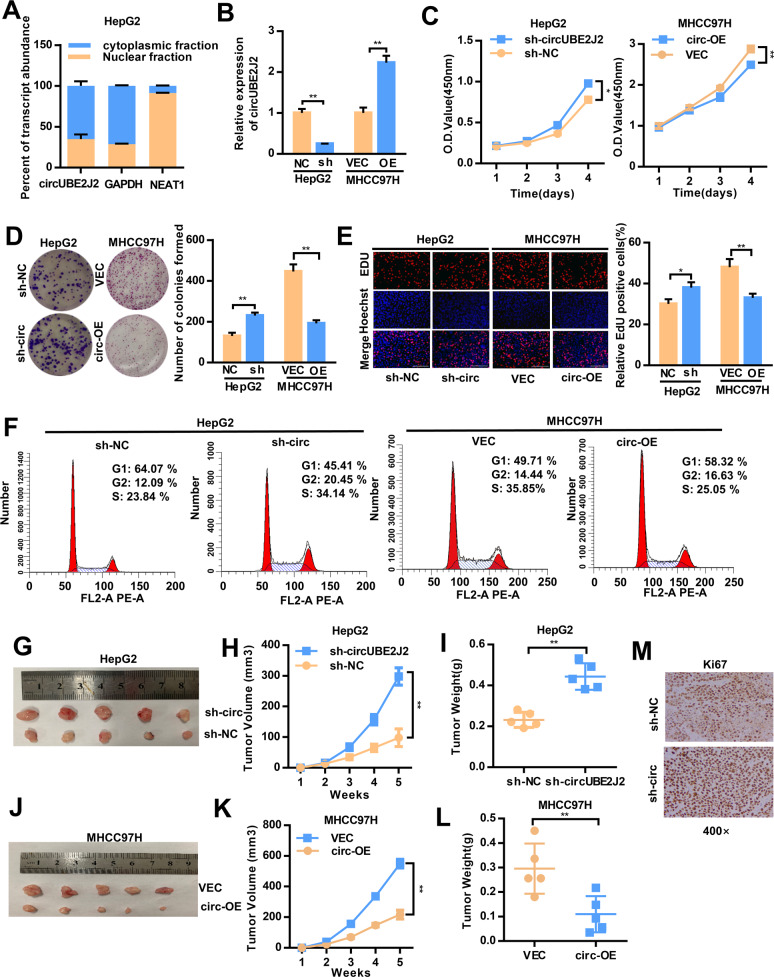
Decrease of circUBE2J2 expression promoted the proliferation of HCC cells in vitro and in vivo. **A** The expression level of circUBE2J2 in the subcellular fractions of HepG2 cells was detected by qRT-PCR. NEAT1 and GAPDH were used as nuclear and cytoplasmic markers, respectively. **B** The overexpression and knockdown efficiency of circUBE2J2 were examined by qRT-PCR. **C–E** CCK-8 (**C**), colony formation assay (**D**), and EDU (**E**) were used to evaluate HCC cells proliferation after circUBE2J2 overexpression or knockdown. Scale bar: white bar, 200 μm. **F** Flow cytometry detection showing the percentages of cells in the G1, S, or G2 phase in both HepG2 and MHCC97H cells. **G–L** HCC cells were subcutaneously injected into nude mice, and tumor growth curves and tumor volume were plotted. Data are presented as mean ± SD; **p < 0.05; **p < 0.01; vs. NC. **M** Expression levels of Ki67 were observed in subcutaneous tumor tissues by IHC. The data are represented as the mean ± SD, *n* = 3. *p < 0.05; **p < 0.01; NS no significance. OE overexpression; sh-circ transfected with lentivirus for circUBE2J2-specific shRNA; sh-NC transfected with control lentivirus.

**Fig. 3:**
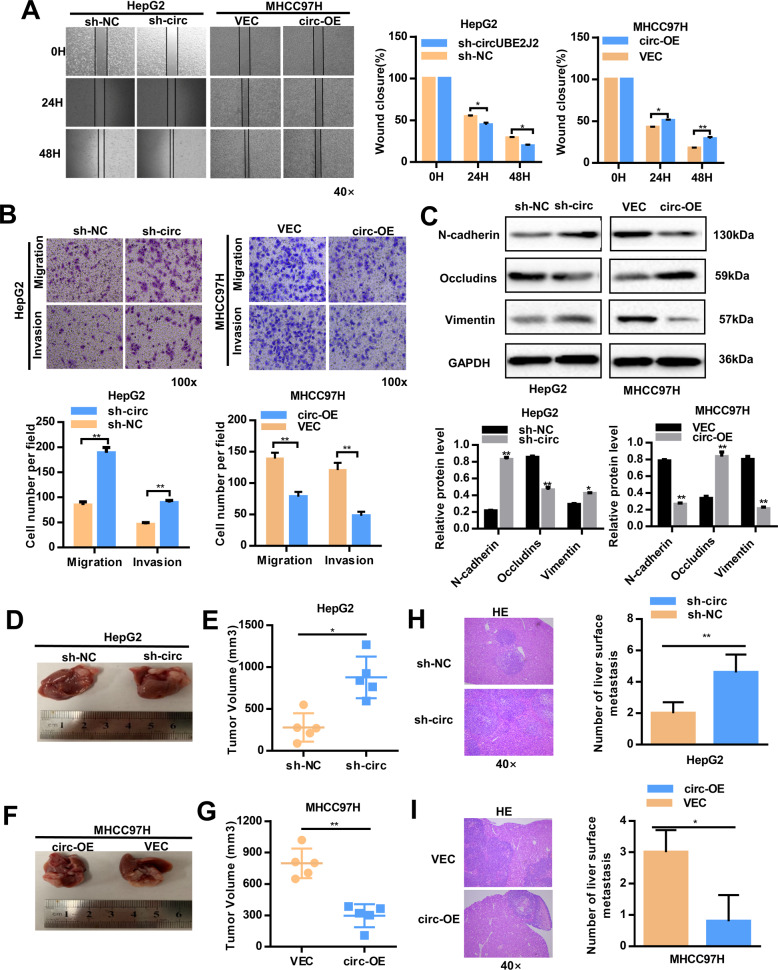
Downregulation of circUBE2J2 promoted HCC migration both in vitro and in vivo. **A** The wound healing of HCC cell. **B** Cell migration and invasion were assessed in both HepG2 and MHCC97H cells using transwell assays. **C** Western blot analysis for the relative levels of EMT-relevant protein expression in HepG2 and MHCC97H cells. **D–I** Tumor growth in HCC cells with the forced expression of circUBE2J2 was investigated by nude mice orthotopic tumor models. In orthotopic model mice, representative images of the liver (**D**, **F**). The size of the largest visible liver nodule was measured (**E**, **G**). Quantifications of visible surface liver metastatic nodules (**H**, **I**). Data are presented as mean ± SD; *p < 0.05; **p < 0.01; NS no significance. OE overexpression; sh-circ transfected with lentivirus for circUBE2J2-specific shRNA; sh-NC transfected with control lentivirus.

First, we determined the intracellular location of circUBE2J2 in HCC cell lines. The results of nuclear cytoplasmic RNA differentiation showed that circUBE2J2 was mostly located in the cytoplasm (Fig. 2A and Additional File 2 Supplementary Fig. S2a). In order to figure out the potential functions of circUBE2J2 in modulating the progression of HCC, a lentivirus expression plasmid (pLKD-CMV-sh-circUBE2J2) was transfected into HLF and HepG2 cells to down-regulate circUBE2J2 levels. We generated circUBE2J2 stably overexpressing MHCC97H cells by transducing them with pLO5-circUBE2J2. The efficiency of transfection was measured and confirmed by qRT-PCR (Fig. 2B and Additional File 2 Supplementary Fig. S2b). Then, qRT-PCR was performed to detect that our transfection had no effects on the UBE2J2 mRNA expression. The expression of UBE2J2 mRNA showed no significant change between sh-circUBE2J2 and shNC, pLO5-circUBE2J2 and vector, confirming the specificity of sh-circUBE2J2 and pLO5-circUBE2J2 (Additional File 2 Supplementary Fig. S2c). The CCK-8 assay, EDU, colony formation assays, cell cycle assay, wound healing assay, and transwell assay revealed a significant promotion in the growth and metastasis of HLF and HepG2 cells after knockdown circUBE2J2 compared with the growth and metastasis of the mock control. Moreover, the overexpressing of circUBE2J2 expression suppressed the growth and metastasis of MHCC97H cells. (Figs. 2C–F, 3A, B and Additional File 2 Supplementary Fig. S2d–j). Given that the EMT process is associated with cancer cell migration and invasion, we tested the impact of altered circUBE2J2 expression on the relative levels of N-cadherin, Occludins, and Vimentin expression in HCC cells by western blot (Fig. 3C and Additional File 2 Supplementary Fig. S2k). The results revealed that circUBE2J2 overexpression significantly increased the levels of Occludins, but decreased N-cadherin and Vimentin expression, while circUBE2J2 silencing had opposite effects.

Next, we further investigated the effects of circUBE2J2 on tumor growth in vivo, HepG2 cells with stable downregulation of circUBE2J2, MHCC97H cells with stable overexpression of circUBE2J2, and their corresponding control were applied to establish subcutaneous and orthotopic implanted intrahepatic HCC models. The size of the tumors was larger in the circUBE2J2-downregulation group compared with the size of the tumors in the mock control after 5 weeks in xenografts. (Figs. 2G–I and 3D, E, and Additional File 3 Supplementary Fig. S3a, b). On the contrary, 5 weeks after HCC cell inoculation, the tumor volume and weight were remarkably decreased in the circUBE2J2-overexpressing group compared with the size of the tumors in the mock control group (Figs. 2J–L and 3F, G and Additional File 3 Supplementary Fig. S3c, d). IHC results suggested that downregulation of circUBE2J2 increased the expression of Ki-67 in xenograft tumor tissues (Fig. 2M). For the orthotopic model, the circUBE2J2 downregulation group had more and larger liver metastatic nodules compared with the control group; the overexpression group was the opposite (Fig. 3H, I). Taken together, these results suggested that circUBE2J2 suppressed tumor growth.

### CircUBE2J2 may function as a sponge for miR-370-5P

**Fig. 4:**
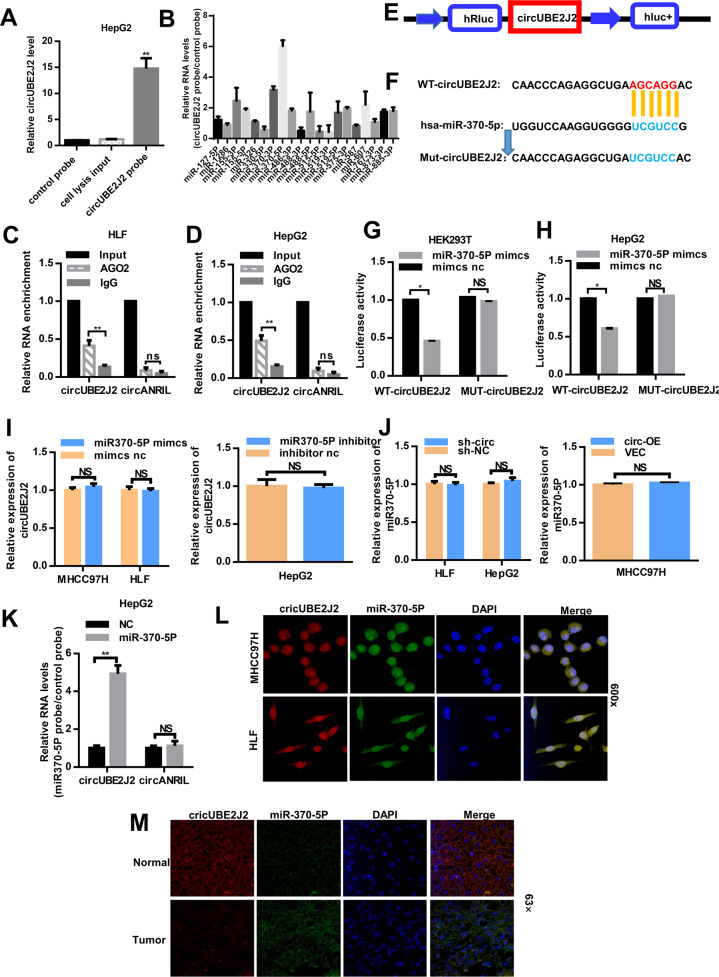
CircUBE2J2 acted as a ceRNA to sponge miR-370-5p in HCC cells. **A** RAP assays were performed using an biotin probe against circUBE2J2 on extracts from HepG2 cells. Relative expression levels of circRNA were evaluated by qRT-PCR. **B** The relative expression level of potential target miRNA of circUBE2J2 was assessed by qRT-PCR. **C**, **D** RIP experiments were performed using an antibody against AGO2 on extracts from HLF and HepG2 cells. **E** Schematic diagram of reporter gene structure. **F** A schematic drawing showing the putative binding sites. The mutant version of circUBE2J2 is presented. **G**, **H** Relative luciferase activity determined 48 h after transfecting HEK293T cells (**G**) and HepG2 cells (**H**) with miR-370-5P mimic/NC or circUBE2J2 WT/Mut. WT wild type, Mut, mutant-type. I The expression of circUBE2J2 in MHCC97H, HLF, and HepG2 cells after transfection with miR-370-5P mimcs or miR-370-5P inhibitor. **J** The expression of miR-370-5P in HepG2, HLF, and MHCC97H cells after transfection with circUBE2J2-OE or circUBE2J2 shRNA. **K** RAP assay were performed using an probe against miR370-5P on extracts from HepG2 cells. Relative expression levels of circRNA were evaluated by qRT-PCR. **L** FISH assay analysis for the co-localization of miR-370-5P and circUBE2J2 in HCC cells. **M** miR-370-5P co-localized with circUBE2J2 in HCC adjacent non-tumor and tumor tissues was detected by FISH. Data are from three independent experiments (mean ± SEM). The data are represented as the mean ± SD, *n* = 3. *p < 0.05; **p < 0.01; NS no significance.

Based on the current research, circRNA is mainly functioned as a miRNA sponge to bind the functional miRNAs and then regulate gene expression in cancer cells [23]. Next, we explored whether circUBE2J2 can bind to certain miRNAs in the progression of HCC. Through circinteractome Program prediction, 22 miRNAs were predicated and listed as possible targets of circUBE2J2 (Additional File 4 Supplementary Fig. S4). In order to screen out functional miRNAs that may interact with circUBE2J2 in HCC cells, we used circUBE2J2-specific biotin-labeled probe to perform RAP assay in HepG2 cells (Fig. 4A). Among the 22 candidate miRNAs predicted by circinteractome, we found a specific enrichment of circUBE2J2 and miR-370-5P as compared to the the other miRNAs (Fig. 4B), determining that miR-370-5P is the circUBE2J2-associated miRNA in HCC cells. Next, we performed RIP assay with an antibody against AGO2 in HLF and HepG2 cells. The results showed that circUBE2J2, but not cANRIL (a circular RNA reported not to bind to AGO2), was significantly enriched by the AGO2 antibody (Fig. 4C, D), suggesting that circUBE2J2 may act as a binding platform for AGO2 and miRNAs. To confirm the prediction, we conducted dual luciferase reporter assay in HEK293T and HepG2 cells. To confirm the bioinformatics prediction, dual-luciferase reporter assay was performed in HEK293T and HepG2 cells. We overexpressed miR-370-5P using RNA mimics in HEK293T and HepG2 cells. We constructed the plasmids of wild type (Wt-circUBE2J2) and mutated (Mut-circUBE2J2) miR-370-5P-binding site into psiCHECK-2 vector (Fig. 4E, F). Wt-circUBE2J2 was found to suppress the reporter activity miR-370-5P mimics group, while there was no significant difference among miR-370-5P mimics and NC group in the relative luciferase activity of Mut-circUBE2J2 group (Fig. 4G, H). However, circUBE2J2 did not show significant changes after increased or reduced miR-370-5P expression in MHCC97H, HLF, and HepG2 cells (Fig. 4I). Neither miR-370-5P showed significant changes after the forced expression or knockdown of circUBE2J2 expression in HCC cells (Fig. 4J). In addition, a pull-down assay using biotin-labeled miR-370-5p showed significant enrichment of circUBE2J2 compared to a negative control (NC) (Fig. 4K). These findings suggest that circUBE2J2 and miR-370-5P may not be degraded by each other. By FISH analysis in HCC cells, HCC tumor and matched non-tumor tissues, we found that circUBE2J2 was co-localized with miR-370-5P in the cytoplasm and this co-localization was decreased in tumor as compared to that in matched non-tumor tissues (Fig. 4L, M). Collectively, these results showed that circUBE2J2 can bind miR-370-5P in the cytoplasm.

### MiRNA-370-5p enhances the malignant behaviors of HCC cells

**Fig. 5:**
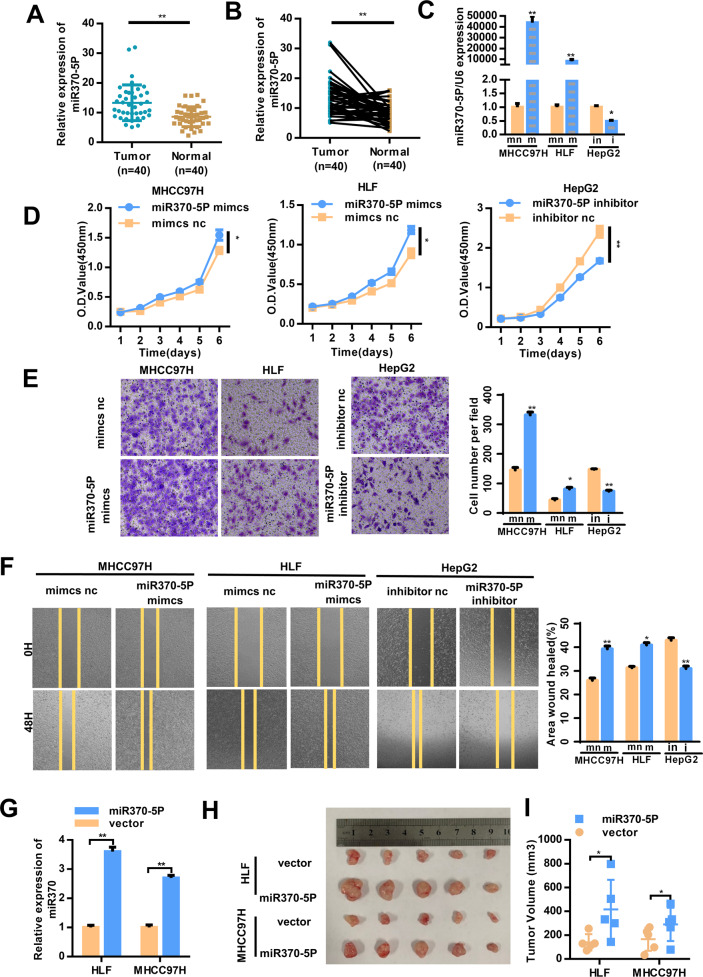
miR-370-5P promoted the progression of HCC cells in vitro and in vivo. **A**, **B** The expression level of miR-370-5P in 40 TNBC tissues and their matched normal adjacent tissues was determined by qRT-PCR. **C** Overexpression and downregulation of miR-370-5P in hepatocellular carcinoma (HCC) cells using a miRNA-370-5P mimic or inhibitor, respectively, as measured by qRT-PCR. **D** miR-370-5P overexpression in MHCC97H and HLF cells promotes cell proliferation as measured by the CCK8 assay; downregulation of miR-370-5P in HepG2 cells inhibits cell proliferation as measured by the CCK8 assay. **E** The transwell assays. **F** The wound healing assays. **G** The expression levels of miR-370-5p were examined by qRT-PCR in HLF and MHCC97H cells with stable miR-370-5p overexpression. **H**, **I** HCC cells were subcutaneously injected into nude mice (**H**) and tumor volume were plotted (**I**). The data are represented as the mean ± SD, *n* = 3. *p < 0.05; **p < 0.01; NS no significance. m miR370-5P mimcs, mn mimcs nc, i miR370-5P inhibitor, in inhibitor nc.

Next, we investigated the biological functions of miR-370-5P. First, qRT-PCR was used to detect its expression profile in 40 pairs of liver cancer tissues and paracancerous non-cancer tissues. The results showed that the miR-370-5P level was significantly higher in HCC tissue compared to non-cancerous tissue (Fig. 5A, B). Then, we enhanced its expression by a miR-370-5P mimic in MHCCC97H and HLF cells, and we downregulated its expression by an miRNA-370-5P inhibitor in HepG2 (Fig. 5C). We performed CCK8 assays, transwell and wound healing assay after manipulating miR-370-5P expression (Fig. 5D–F). Compared with the controls, miRNA-370-5P overexpression significantly enhanced the proliferation, and migration of MHCC97H and HLF cells, while miRNA-370-5P silencing attenuated the proliferation and migration of HepG2 cells in vitro. We generated miRNA-370-5P stably overexpressing cell lines (Fig. 5G). HCC cells were injected to nude mice subcutaneously to investigate the role of miR-370-5P. The volume of subcutaneous tumors significantly increased after overexpressing in MHCC97H and HLF cells (Fig. 5H, I). Taken together, these results suggested that miR-370-5P promoted the malignant behaviors of HCC cells.

### MiRNA-370-5p targets the KLF7 expression

**Fig. 6:**
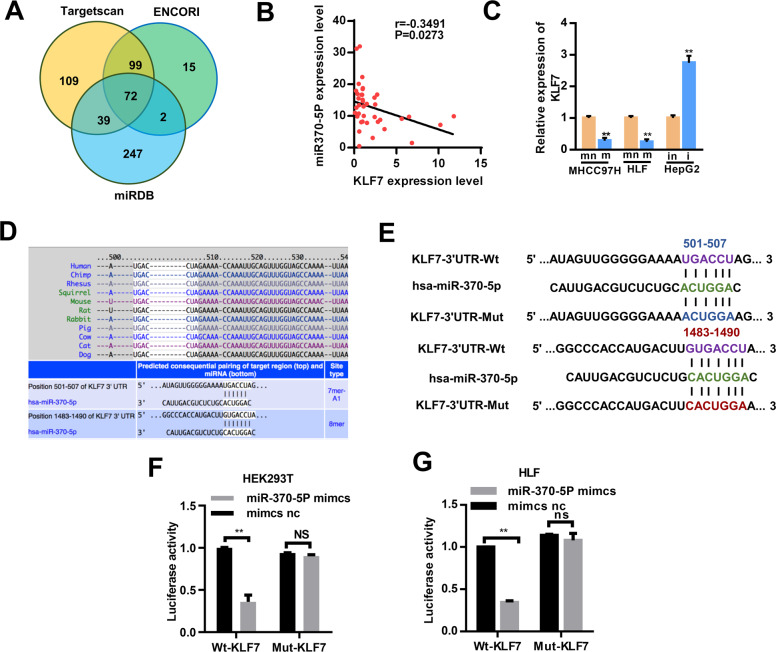
KLF7 is a direct target of miR-370-5p. **A** Venn diagram analyses of three independent databases reveals 72 possible targets of miR370-5P. **B** Pearson’s correlation analysis showed that miR-370-5P was inversely correlated with KLF7 mRNA levels. **C** Overexpression and downregulation of miR-370-5P in MHCC97H,HLF and HepG2 cells changes the KLF7 mRNA level as measured by qRT-PCR. **D** Bioinformatics predicted binding sites of miR-370-5P within KLF7 were shown. **E** A schematic drawing showing the putative binding sites. The mutant version of KLF7 is presented. **F**, **G** Relative luciferase activity determined 48 h after transfecting HEK293T cells (**D**) and HLF cells (**E**) with miR-370-5P mimic/NC or KLF7 WT/Mut. Data are presented as means ± SD, *n* = 3. **p < 0.05; **p < 0.01. WT wild-type, Mut mutant-type, m miR370-5P mimcs, mn mimcs nc, i miR370-5P inhibitor, in inhibitor nc.

Next, the potential target genes of miRNA-370-5p were predicted by bioinformatics by using TargetScanHuman (www.targetscan.org), ENCORI (http://starbase.sysu), and miRDB (http://mirdb.org/) (Fig. 6A). We selected 32 genes most likely to be related to tumors from 72 candidate genes for qRT-PCR verification after reviewing the relevant literature (Additional File 5 Supplementary Fig. S5a–h). However, we detected significant changes in the expression levels of these mRNAs in MHCC97H and HLF cells transfected with a miR-370-5P mimic and found that KLF7 might be a potential target gene of miR-370-5p. While miR-370-5p overexpression significantly decreased KLF7 mRNA transcripts in MHCC97H and HLF cells, miR-370-5p silencing increased KLF7 mRNA transcripts in HepG2 cells (Fig. 6B, C). Moreover, there was an inverse association between the miR-370-5P and KLF7 expression levels in 40 cases of HCC tissues (Fig. 6D). Furthermore, the potential binding sites of miR-370-5P and KLF7 were predicted by TargetScan (Fig. 6E). To verify the prediction, we used dual luciferase reporter assay to verify whether miR-370-5p could combine with the KLF7 mRNA 3′UTR. The KLF7–3′UTR-Wt and KLF7–3′UTR-Mut were cloned into luciferase reporter vector psiCHECK-2 (Fig. 6F). We then transfected this reporter vector into HLF and HEK293T cells combined with a miR-370-5P mimic or not, respectively. Results showed that the relative luciferase activity of the KLF7–3′UTR-Wt group significantly decreased in the miR-370-5p mimics group compared with that in the NC group, but these effects disappeared in the KLF7–3′UTR-Mut group (Fig. 6G, H). These findings indicate a direct interaction between miR-370-5P and KLF7 mRNA in HCC cells.

### KLF7 attenuates the malignant behaviors of HCC cells

**Fig. 7:**
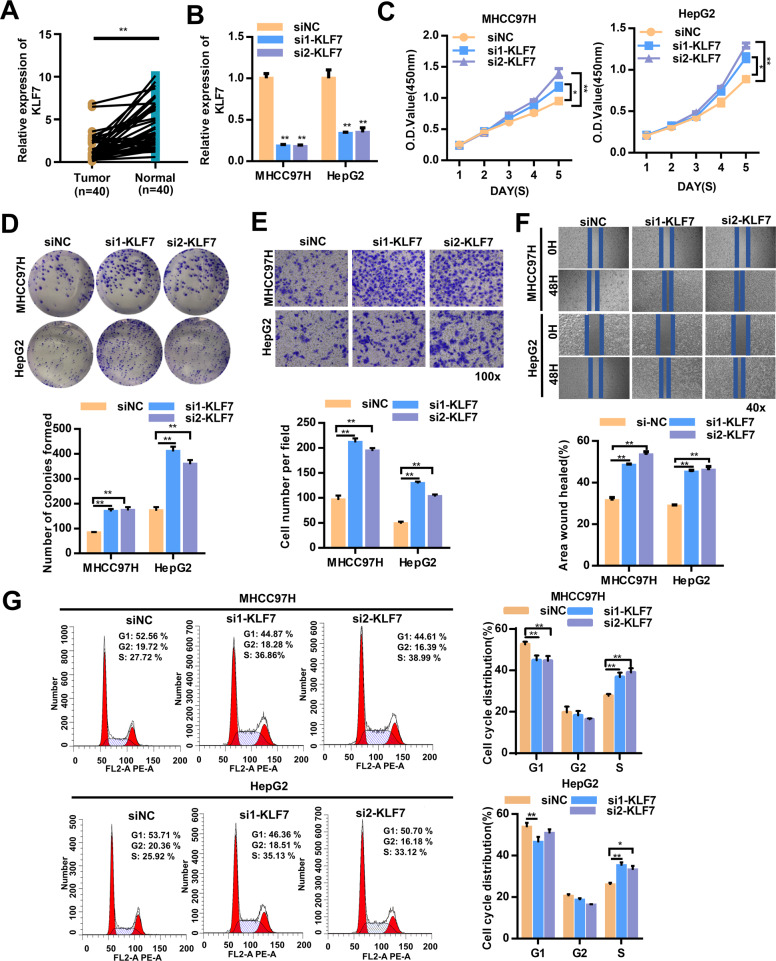
KLF7 inhibits the proliferation, wound healing, and migration of MHCC97H and HepG2 cells in vitro. **A** qRT-PCR detection show the differential expression of KLF7 in 40 paired HCC tissues. **B** qRT-PCR detection showing the expression of KLF7 in both MHCC97H and HepG2 cells after transfection with siRNA against KLF7 or negative control. **C** CCK8 assays were used to evaluate cell proliferation. **D** Colony formation assay showing proliferation in both MHCC97H and HepG2 cells. **E** The migration of HCC cells. **F** The wound-healing assays. **G** Flow cytometry detection showing the percentages of cells in G1, S, or G2 phase in both MHCC97H and HepG2 cells. Data are presented as mean ± SD, *n* = 3. *p < 0.05, **p < 0.01; NS no significance.

Next, we investigated the biological function of KLF7 in HCC. We first used qRT–PCR to determine the expression level of KLF7 in 40 cases of HCC tissues. Our results showed that the KLF7 level was significantly higher in adjacent non-cancerous tissues compared to HCC tissue (Fig. 7A). MHCC97H and HepG2 cells were transfected with siRNA against KLF7 or control siRNA. The effects of KLF7 knocking down in HCC cells were measured by qRT-PCR (Fig. 7B). The results of CCK-8 assay, colony formation assays,, wound healing assay, transwell assay, and cell cycle assay suggested that knockdown of KLF7 promoted cell proliferation and migration (Fig. 7C–G). Thus, KLF7, like the circUBE2J2, attenuated the malignant behaviors of HCC cells.

### CircUBE2J2 inhibited HCC progression through the circUBE2J2/miR-370-5p/KLF7 axis

**Fig. 8:**
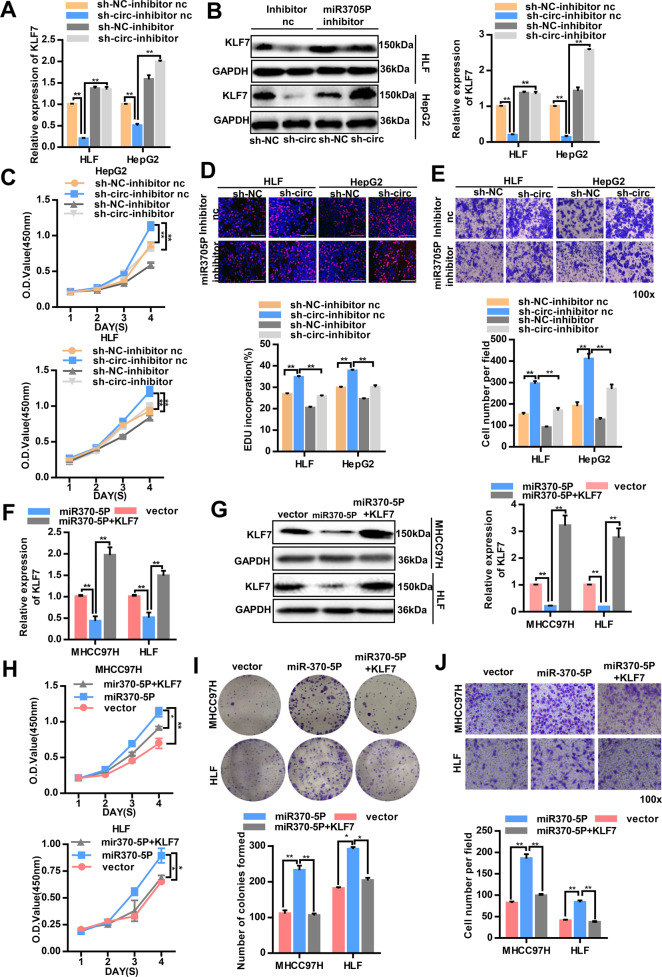
CircUBE2J2 inhibited HCC progression through the circUBE2J2/miR-370-5p/KLF7 axis. **A** KLF7 expression in the mRNA level was measured by qPCR in HCC cells after circUBE2J2 knockdown. **B** After circUBE2J2 knocking down, miR-370-5P inhibitor or inhibitor nc were added to HLF and HepG2 cell culture. The KLF7 protein expression level was analyzed by western blot. **C** The cell proliferation in HCC cells with the reduced expression of miR-370-5P was assessed by a CCK-8 assay. **D** The proliferation ratio of HCC cells was measured by EdU incorporation. **E** The migration abilities in HCC cells with the reduced expression of miR-370-5P were evaluated via a transwell assay. **F** After miR-370-5P overexpression, KLF7 overexpression vector was added to MHCC97H and HLF cell culture. KLF7 expression in the mRNA level was measured by qPCR. **G** The KLF7 protein expression level was analyzed by western blot. **H** The cell proliferation in HCC cells was assessed by a CCK-8 assay. **I** Colony formation assays show the proliferation. **J** Transwell migration assays. Data are presented as mean ± SD, *n* = 3. *p < 0.05, **p < 0.01; NS no significance. sh-circ transfected with lentivirus for circUBE2J2-specific shRNA; sh-NC transfected with control lentivirus.

According to our previous experiments, downregulation of circUBE2J2 markedly promoted HCC cell proliferation and migration. To identify whether circUBE2J2 exerts its biological functions through the miR-370-5P/KLF7 axis, we performed rescue experiments using miR-370-5p inhibitors to detect whether the tumor-promoting effect of knocking down circUBE2J2 could be blocked by inhibiting miR-370-5p. Next, we explored whether the silencing of miR-370-5p could affected the proliferation and migration of HCC cells that knocked down circUBE2J2. The knockdown efficiency was detected by qRT-PCR and western blot analysis (Fig. 8A, B). As expected, the silencing of miR-370-5P increased KLF7 mRNA and protein expression both HLF and HepG2 cell lines with circUBE2J2 downregulated. Furthermore, in the CCK-8, EDU, transwell assays, the silencing of miR-370-5P significantly suppressed the proliferation, and migration abilities of HLF and HepG2 cells after knocking down circUBE2J2 (Fig. 8C–E).

Next, we further sought to explore whether the biological functions of miR-370-5P in HCC cells could be reversed by KLF7 overexpression. KLF7-expressing plasmid or vector were added to MHCC97H and HLF cells that overexpressed miR-370-5P. Next, qRT-PCR and western blot analyses validated that the expression of KLF7 was reduced by miR-370-5P (Fig. 8F, G). CCK8 (Fig. 8H) assays and colony formation (Fig. 8I) showed that overexpressing miR-370-5P promoted proliferation of MHCC97H and HLF cells. Overexpressing KLF7 rescued the proliferation ability of HCC cells because miR-370-5P could not interact with exogenous KLF7, which lacked a 3′UTR after transcription. Transwell assays to measure cell migration (Fig. 8J) also found that overexpressing KLF7 rescued the migration ability of both MHCC97H and HLF cells that overexpressed miR-370-5P. In summary, these results indicated that circUBE2J2 inhibit HCC cell progression might through the circUBE2J2/miR-370-5P/KLF7 axis.

## Discussion

HCC is one of the leading causes of malignancy in humans, and has high morbidity and mortality rates [24]. Circular RNAs (circRNAs), microRNAs (miRNAs), and other noncoding RNAs can regulate cell activities [25]. Various treatment methods such as surgical resection, liver transplantation, and radiofrequency ablation have been continuously improved; the therapeutic effect of HCC has improved. However, due to the high recurrence rate and metastasis rate after HCC surgery, the overall prognosis of HCC patients is still unsatisfactory [5]. Therefore, the mechanisms of HCC development require further investigation. Our high-throughput sequencing study found that circUBE2J2 expression was decreased in HCC tissues. Our clinical study have confirmed that the expression of circUBE2J2 was significantly associated with smaller tumor size, multiple nodules, and invasion in this population. Further validation in 75 pairs of HCC and non-tumor liver tissues showed that the down-regulated expression of circUBE2J2 was associated with poorer overall survival. The expression of circUBE2J2 was down regulated, suggesting that circUBE2J2 might be used as a tumor suppressor to reduce the malignant behavior of liver cancer. In fact, we found that overexpression of circUBE2J2 can attenuate the malignant behavior of liver cancer cells in vivo and in vitro, inhibit the growth of liver cancer cells in vivo. Therefore, these findings indicate the role of circUBE2J2 in advancing HCC. Therefore, circUBE2J2 may be a therapeutic target for liver cancer, and our research results may provide a new idea for the pathogenesis of liver cancer. The nuclear–cytoplasmic RNA fractionation and FISH assay revealed that circUBE2J2 mostly located in the cytoplasm.

It is well known that a circRNA can bind to its targeted miRNAs and act as a ceRNA to sponge these miRNAs and inhibit their activity [26], while miRNAs bind to the 3′UTR of mRNAs to suppress their translation, and promote their degradation [27]. circRNA through sponging the miRNAs would enhance the miRNA-targeted gene expression. CircMTO1 inhibits HCC growth by the sponge activity on miR-9 and upregulation of p21 expression [28]. circRNA SRY was reported to be a tumor-related molecule in colorectal cancer by absorbing miR-138 [29]. In this study, by performing RAP and RIP using circUBE2J2-specific probe, we found that miR-370-5p was the circUBE2J2-binding miRNA, which was also confirmed by luciferase reporter gene assay. To the best of our knowledge, this was the first report on upregulated miR-370-5P expression in HCC, which extended previous reports of miR-370-5p in other cancers. Other studies have found that miR-370-5p expression can affect the progression of breast cancer [30], lung cancer [31], and ovarian cancer [32]. MiR-370-5p expression in HCC tissues was significantly higher than adjacent tissues of the carcinoma. These results indicated that miR-370-5p may have a cancer-promoting effect in liver cancer. From functional studies in vitro and in vivo, we found that the up-regulation of miR-370-5p significantly enhanced the proliferation and metastasis of HCC cells, while the down-regulation of miR-370-5p showed an opposite trend. Therefore, we speculated that circUBE2J2 may play a tumor suppressor effect in HCC by down-regulating the expression of miR-370-5P. Next, we attempted to find the potential target genes of miR-370-5P by using bioinformatics analysis. The results of qRT-PCR and dual-luciferase reporter assay confirmed that KLF7 was a direct target of miR-370-5p. We found that overexpression of miR-370-5p significantly reduced the mRNA of KLF7, and the expression of miR-370-5P and KLF7 was negatively correlated in our clinical HCC tissues, suggesting that miR-370-5p is an important negative regulator of KLF7. KLF7 is a member of the Kruppel-like transcriptional regulator family. Members of the KLF family have established roles in tumor cell proliferation, differentiation, survival, cell fate, stress response, and the tumor-initiating properties of cancer stem-like cells [33]. Knockdown of KLF7 inhibits the expression of IFN-stimulated genes, which are necessary for KLF7-mediated PDAC tumor growth and metastasis [34]. KLF7 has not been reported in HCC yet. Our study found that silencing circUBE2J2 decreased KLF7 expression by enhancing miR-370-5P expression. Downregulation of circUBE2J2 or up-regulation of miR-370-5P and silencing of KLF7 can promote the invasion and proliferation of HCC cells. Next, inhibition of miR-370-5P reversed the effect of circUBE2J2 knockdown. Exogenously overexpressing KLF7 rescued the proliferation and migration abilities of HCC cells after upregulating miR-370-5P. These findings suggested that circUBE2J2 inhibits HCC progression via sponging miR-370-5P, which enhances KLF7 expression. And our data indicated that circUBE2J2 may have considerable potential as a prognosis predictor and therapeutic target for HCC.

## Conclusion

In summary, these data illustrated that circUBE2J2 expression decreased in HCC and was closely correlated to HCC development and occurrence. HCC patients with lower expression of circUBE2J2 had a poorer overall survival rate. We showed that circUBE2J2 directly targets miR-370-5P, which led to decreased miR-370-5P expression. Decreased circUBE2J2 promote HCC progression by increasing miR-370-5P levels, which led to decreased KLF7 expression. Thus, our findings provide a novel target for HCC treatment that warrants further investigation.

## Supplementary information

Supplement table1

Supplementary Figure S1

Supplementary Figure S2

Supplementary Figure S3

Supplementary Figure S4

Supplementary Figure S5

## Data Availability

All the data and materials supporting the conclusions were included in the main paper.
