# Supplementary figures and images for: Circular RNA circUBE2J2 acts as the sponge of microRNA-370-5P to suppress hepatocellular carcinoma progression
Source: Cell Death Dis. 2021 Oct 22;12(11):985. doi: 10.1038/s41419-021-04269-4 (PMC8536678; doi:10.1038/s41419-021-04269-4)

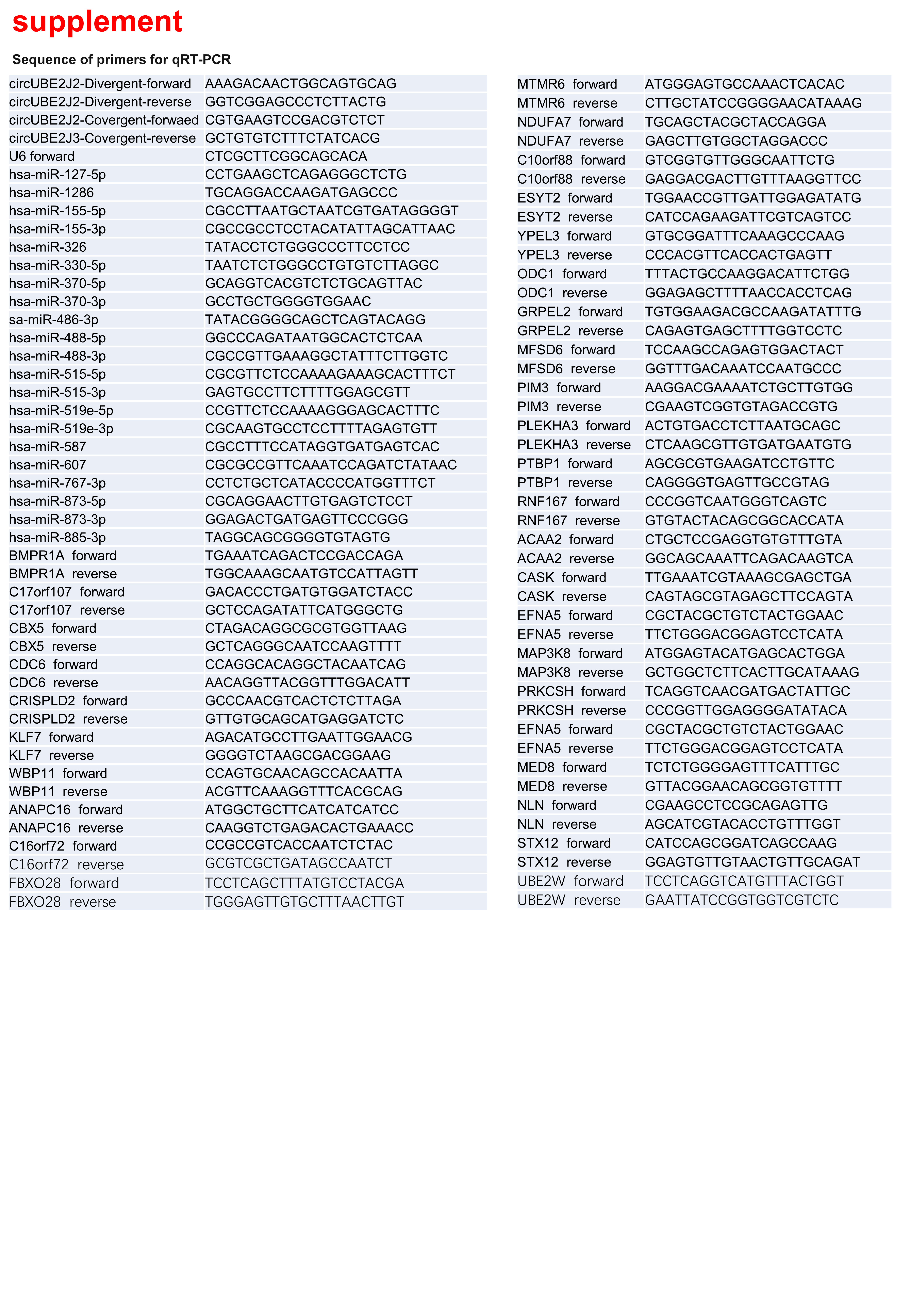

Supplement: Supplementary file 1 — Supplement table1 [file 41419_2021_4269_MOESM1_ESM.tif]

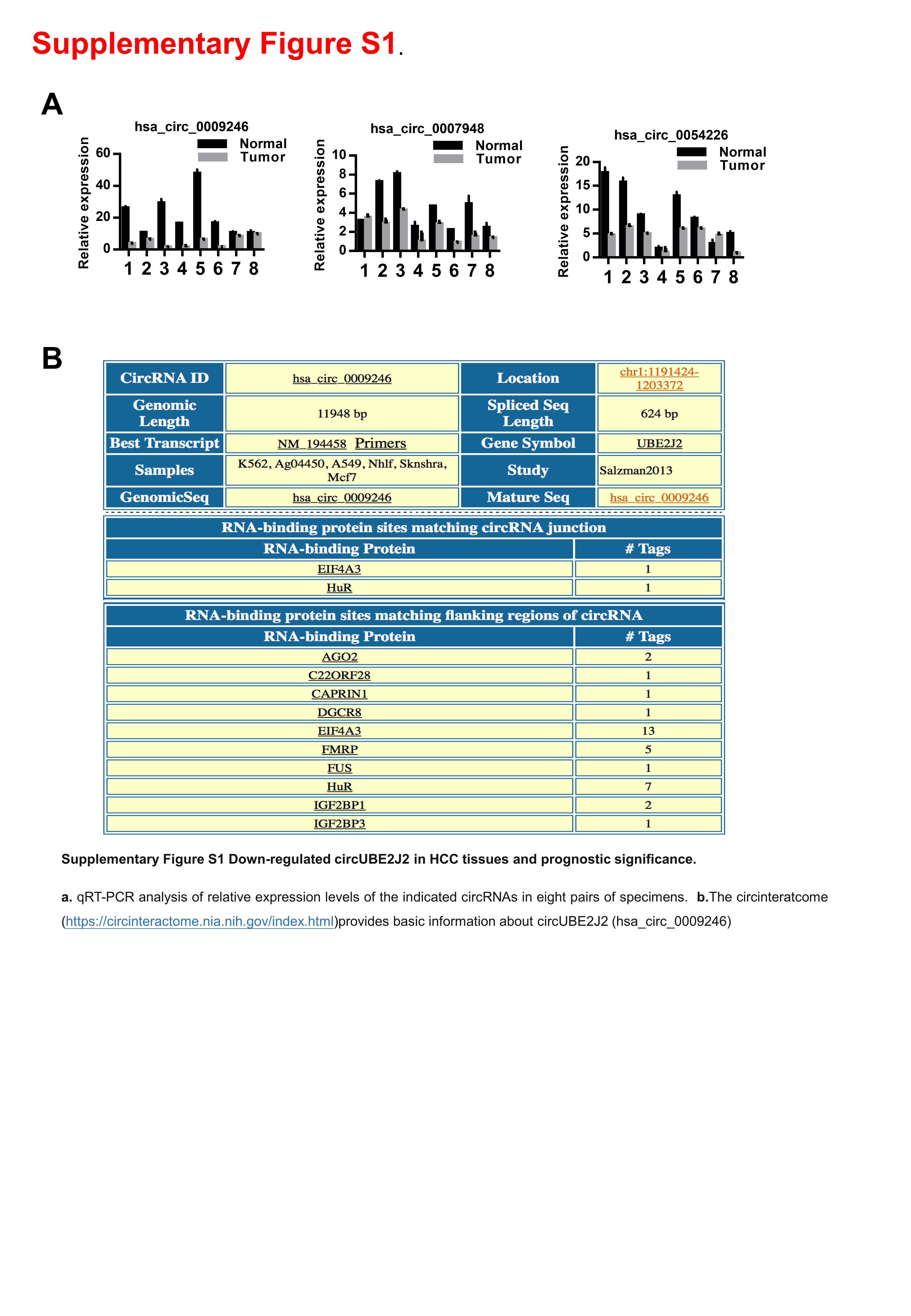

Supplement: Supplementary file 2 — Supplementary Figure S1 [file 41419_2021_4269_MOESM2_ESM.tif]

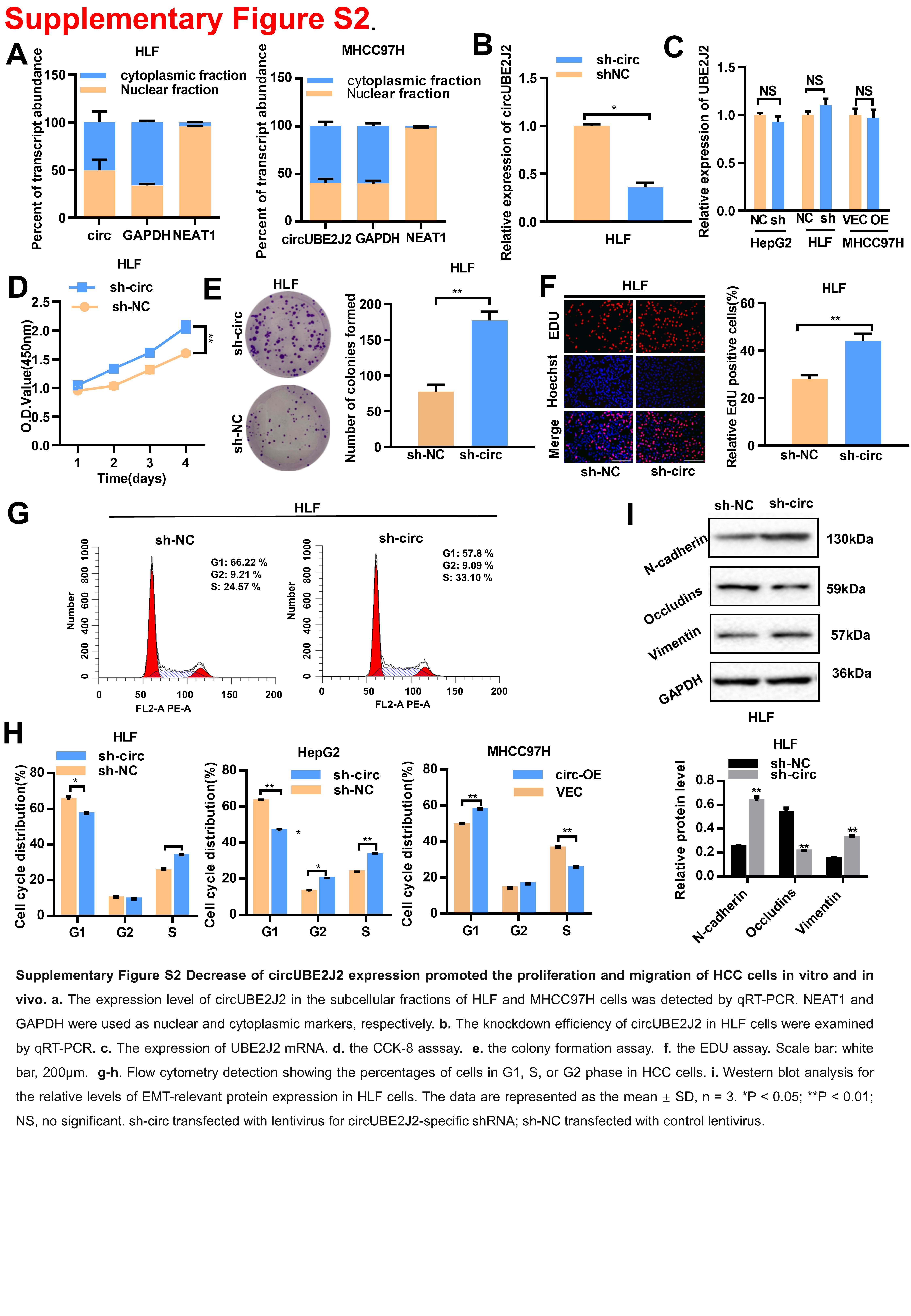

Supplement: Supplementary file 3 — Supplementary Figure S2 [file 41419_2021_4269_MOESM3_ESM.tif]

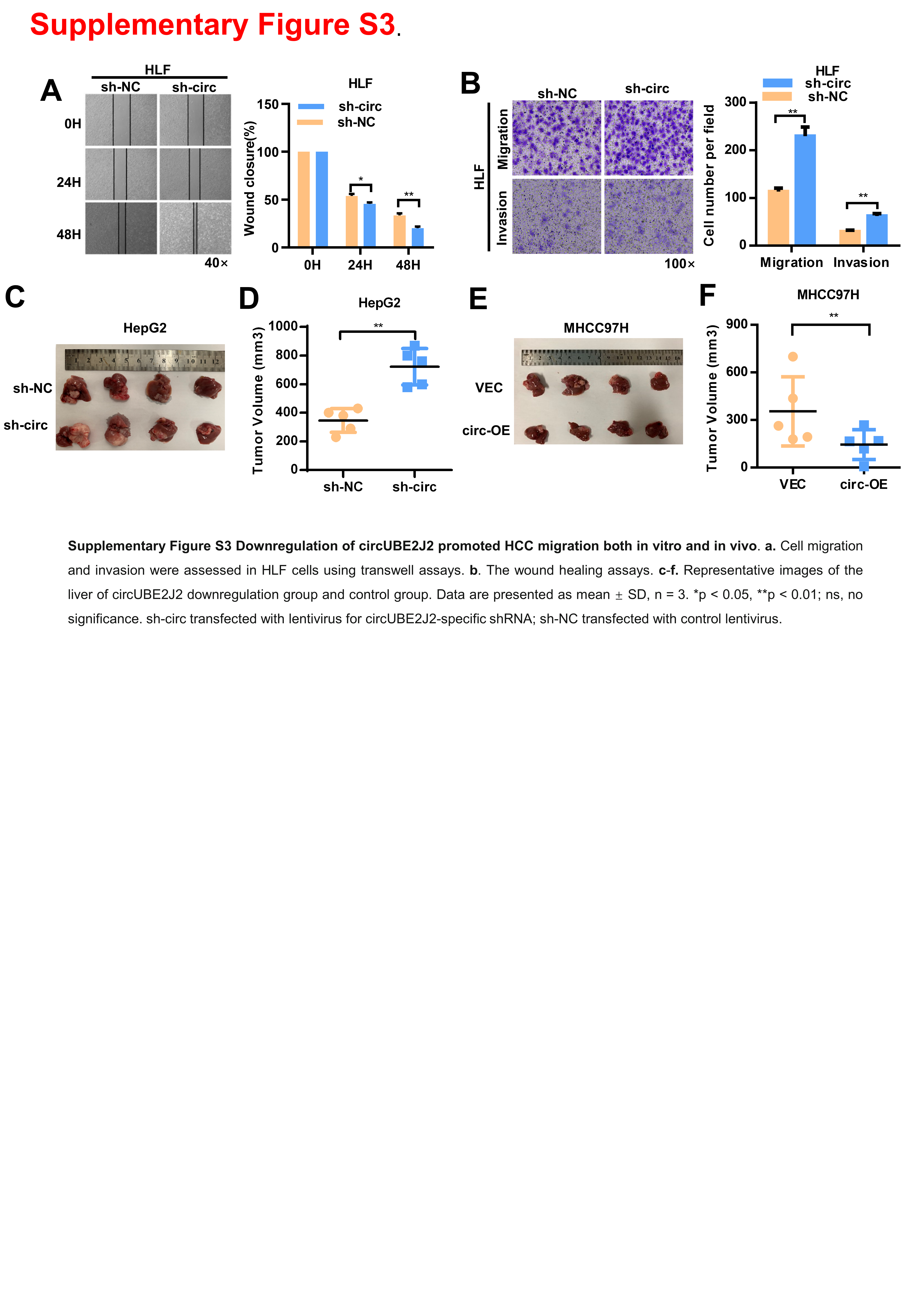

Supplement: Supplementary file 4 — Supplementary Figure S3 [file 41419_2021_4269_MOESM4_ESM.tif]

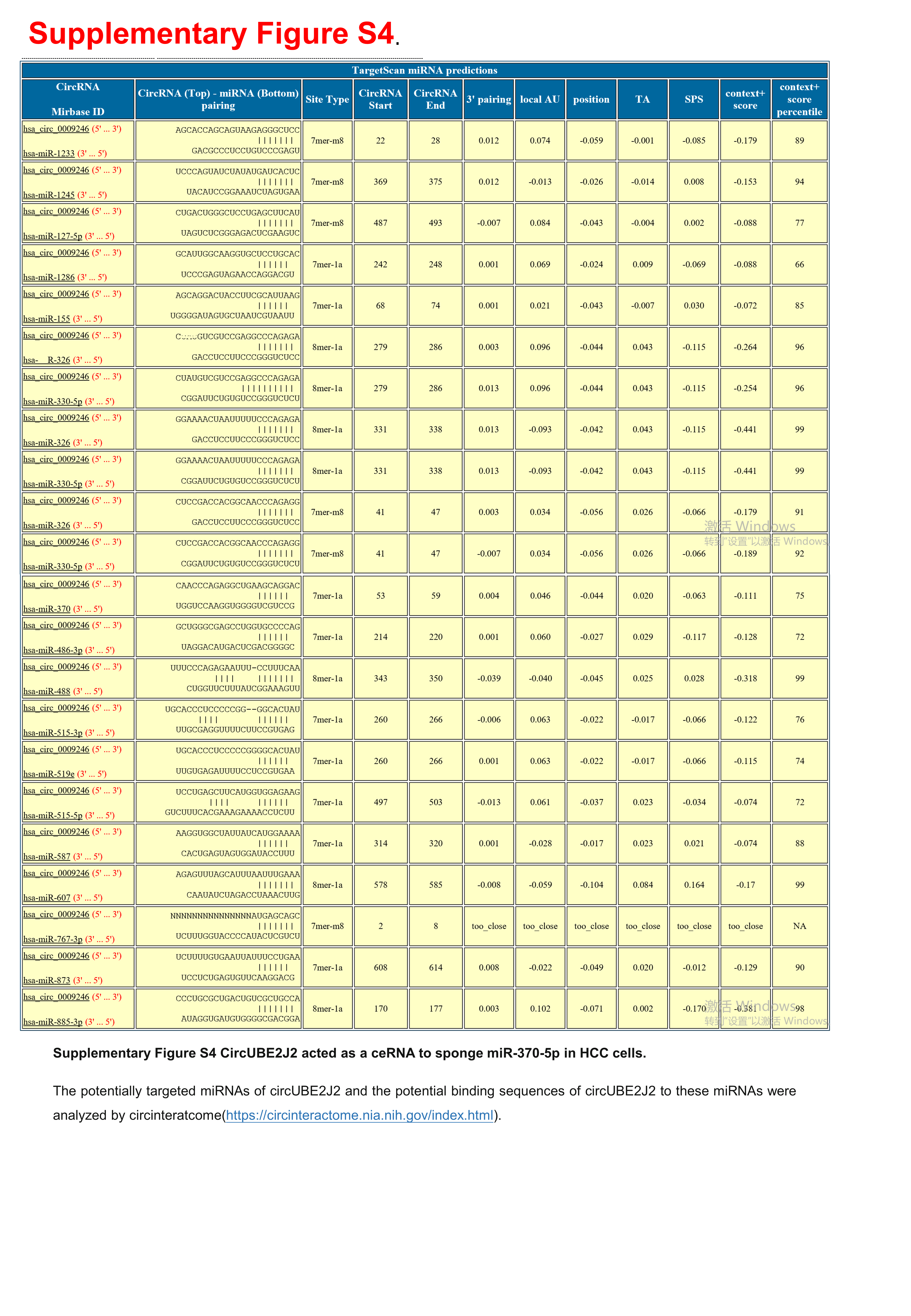

Supplement: Supplementary file 5 — Supplementary Figure S4 [file 41419_2021_4269_MOESM5_ESM.tif]

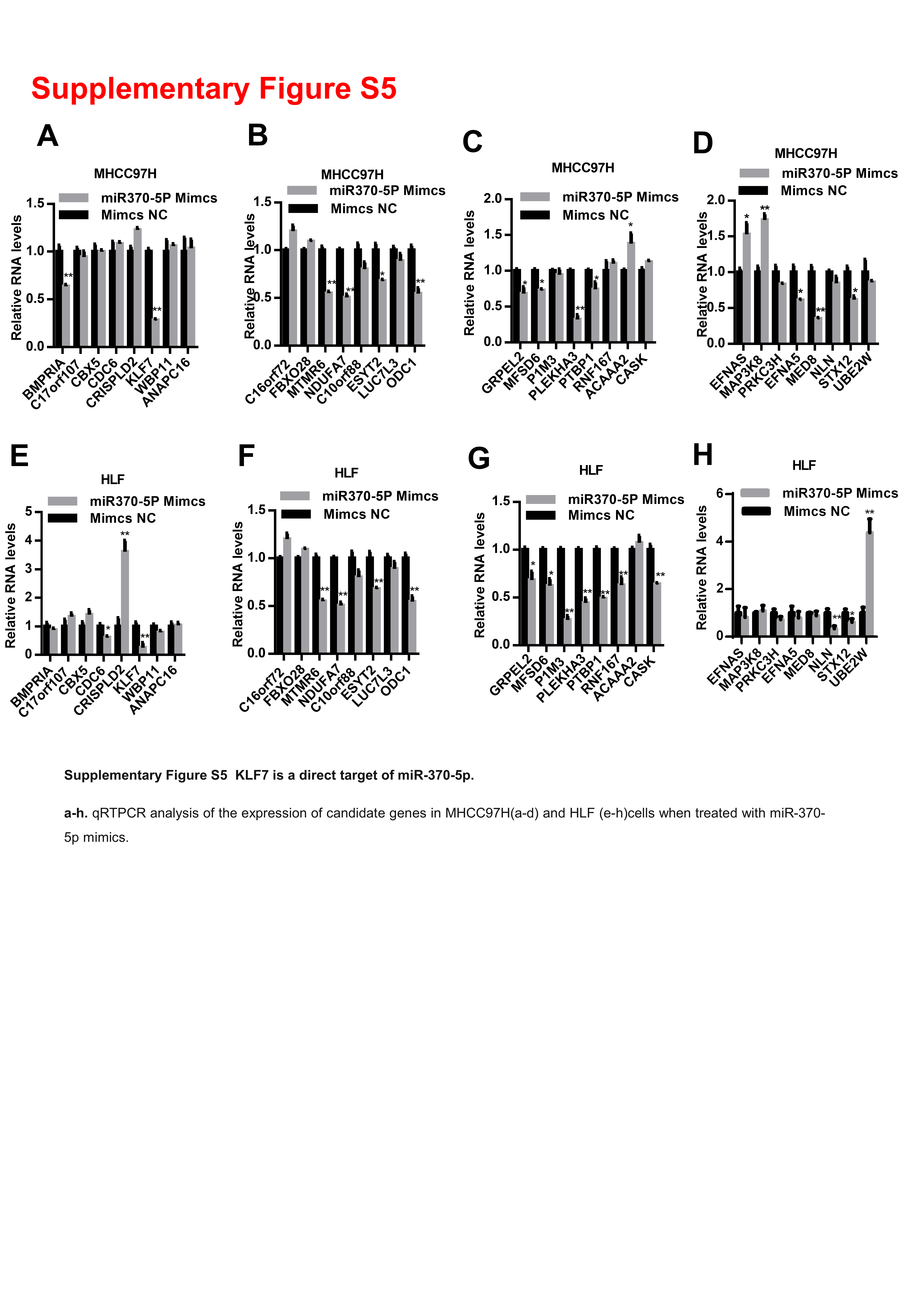

Supplement: Supplementary file 6 — Supplementary Figure S5 [file 41419_2021_4269_MOESM6_ESM.tif]
